# Establishment of three cell lines from Chinese giant salamander and their sensitivities to the wild-type and recombinant ranavirus

**DOI:** 10.1186/s13567-015-0197-9

**Published:** 2015-06-12

**Authors:** Jiang-Di Yuan, Zhong-Yuan Chen, Xing Huang, Xiao-Chan Gao, Qi-Ya Zhang

**Affiliations:** State Key Laboratory of Freshwater Ecology and Biotechnology, Institute of Hydrobiology, Chinese Academy of Sciences, Graduate University of Chinese Academy of Sciences, Wuhan, 430072 China

## Abstract

Known as lethal pathogens, Ranaviruses have been identified in diseased fish, amphibians (including Chinese giant salamander *Andrias davidianus*, the world’s largest amphibian) and reptiles, causing organ necrosis and systemic hemorrhage. Here, three Chinese giant salamander cell lines, thymus cell line (GSTC), spleen cell line (GSSC) and kidney cell line (GSKC) were initially established. Their sensitivities to ranaviruses, wild-type *Andrias davidianus* ranavirus (ADRV) and recombinant *Rana grylio* virus carrying EGFP gene (rRGV-EGFP) were tested. Temporal transcription pattern of ranavirus major capsid protein (MCP), fluorescence and electron microscopy observations showed that both the wild-type and recombinant ranavirus could replicate in the cell lines.

## Introduction, methods and results

The Chinese giant salamander *Andrias davidianus* is the largest extant species of amphibian in the world and is often crowned as a living fossil [1]. The wild Chinese giant salamanders are on the verge of extinction, arousing wide research interest in its conservation [2]. Unfortunately, the prevalence of iridoviruses (including ranavirus and lymphocystis virus) infections is increasingly associated with major declines of amphibian species worldwide, causing extensive damage to aquaculture [3-7]. Ranaviruses are icosahedral DNA viruses, and are recognized as major viral pathogens of ectothermic vertebrates, namely fish, amphibians and reptiles [8-10]. Ranaviruses have been identified in diseased Chinese giant salamanders, such as *Andria davidianus* ranavirus (ADRV), which pose a serious threat to its population [11-14]. Another ranavirus strain, *Rana grylio* virus (RGV) is a pathogenic agent that causes lethal disease in cultured frogs (*Rana grylio*), which is the first isolated ranavirus in China [15]. In recent years, several recombinant RGV, including ΔTK-RGV, Δ67R-RGV and i53R-RGV-lacIO were constructed in our laboratory [16-18]. Recombinant virus technology has provided novel approaches for unraveling virus-host interactions and host antiviral immune response [19].

Cell culture is essential for virus replication and isolation, because they are obligate intracellular agents [20]. A cell culture-based technique has been popular for studying virus infection mechanisms, viral gene functions and virus-host interactions in aquaculture animals, and it is regarded as the “gold standard” for detection and diagnosis of viral pathogens [21,22]. Besides, the cultured cells have been used to replace live animals in testing agents in virology, pharmacology, toxicology, etc. [23]. However, in contrast to fish, from which various cell lines have been established [24], few permanent cell lines have been established from the Amphibian, Order Caudata [25,26]. To our knowledge, no cell line from Chinese giant salamanders is currently available. Therefore, we attempted to establish different cell lines from the Chinese giant salamander and to investigate their sensitivities to the wild-type and recombinant ranavirus.

In the present study, one-year-old healthy Chinese giant salamanders were anesthetized, killed and wiped with 75 v/v ethanol, and the thymus, spleen and kidney tissues were removed for primary culture. All animal procedures were conducted in accordance with the recommendations in the Regulations for the Administration of Affairs Concerning Experimental Animals of China, and all efforts were made to minimize suffering.

**Figure 1:**
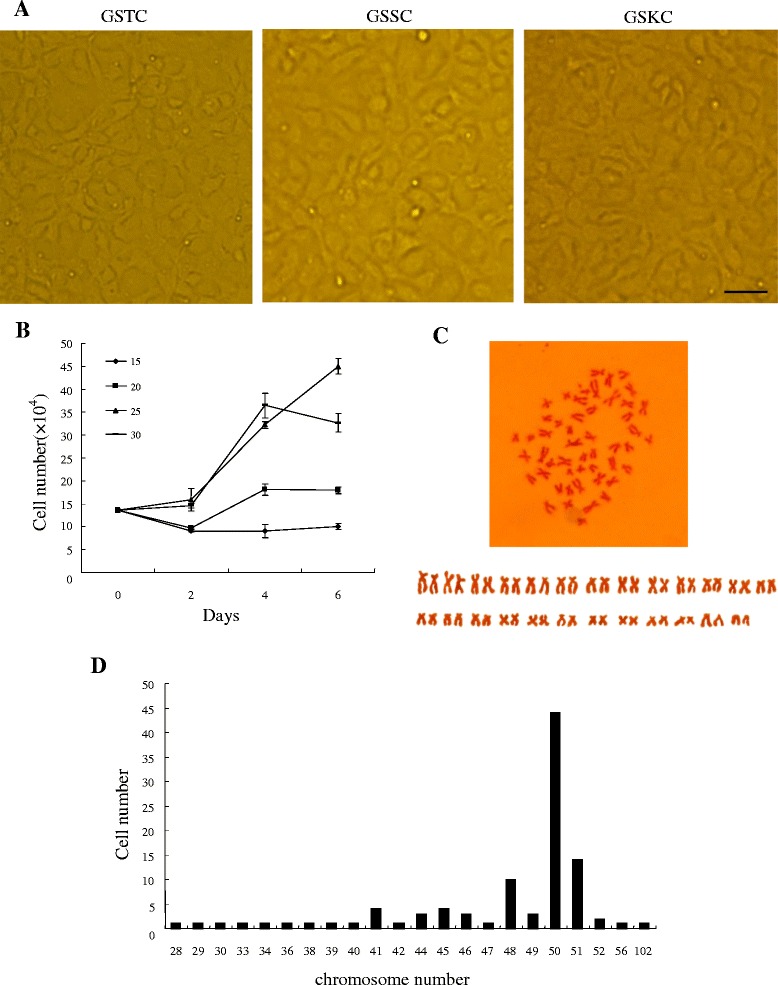
**Characteristics of GSTC, GSSC and GSKC cells.** **(A)** The monolayer of the GSTC, GSSC and GSKC cells exhibited epithelial-like morphology. Bar = 100 μm. **(B)** Growth curves of GSTC cells at different temperatures (15, 20, 25 and 30 °C). **(C)** Metaphase chromosome spread (upper panel) and diploid karyotype (under panel). **(D)** Chromosome number distribution of GSTC cells.

The tissues were immersed in 70% v/v ethanol for 30 s, washed three times with sterile phosphate-buffered saline and minced thoroughly with scissors, followed by incubation in glass plates with TC199 medium containing 1000 units mL^−1^ penicillin (Sigma) and 1000 μg/mL streptomycin (Sigma) for 1 h. The minced tissues were attached to 25 cm^2^ cell culture flasks with 4 mL of TC199 medium supplemented with 20% fetal bovine serum (FBS), 100 units/mL penicillin and 100 μg/mL streptomycin by a tissue explant method. The cell cultures were maintained at 25 °C, and one half of the growth medium was changed every 4 days. The primary cells emigrated from tissues after a month, and were heterogeneous in morphology. The serum concentration was changed to 10% after the cells reached 80% confluence. Thus the Chinese giant salamander thymus cell line (GSTC), spleen cell line (GSSC), and kidney cell line (GSKC) were established after subcultured for more than 60 passages. As shown in Figure 1A, three cell lines all exhibited epithelial-like morphology. The three cell lines at passages of 10, 24 and 58 were cryopreserved and recovered successfully, respectively, according to methods previously described [27]. Briefly, after being subcultured for three days, the cells were trypsinized and suspended in 3 mL TC199 medium containing 10% dimethyl sulfoxide and 20% FBS. The cell suspensions were transferred to a 2-mL cryovial, kept in a foamy box and stored in −80 °C quickly. GSTC was taken as a model for further characterization. The effects of incubation temperature on GSTC cell growth were studied at the 45th passage level. GSTC cells were inoculated into a 24-well plate at a density of 1.3 × 10^5^ cells/well and incubated at 15, 20, 25 and 30 °C, respectively. Triplicate wells of cells at each temperature were trypsinized and counted using a hemocytometer every two days. Figure 1B shows that GSTC cells grew best at 30 °C in the first 4 days, and 5 days later the highest growth rate was obtained at 25 °C. The other two cell lines (GSSC and GSKC) grew best at 25 °C too. Chromosome preparations were obtained from the cells (such as GSTC, at passage 45) using a method described previously [24]. Hundred photographed cells at metaphase were counted and the karyotype was analyzed. The chromosome numbers varied from 28 to 102, with a distinct peak at 50. The metaphase displayed the karyotype morphology (Figure 1C) consisting of 12 pairs of metacentrics (m), 11 pairs of submetacentrics (sm), 1 pair of telocentrics (t) and 1 pair of subtelocentrics (st): (2n = 24 m + 22sm + 2 t + 2st). The chromosome analysis revealed that GSTC has a diploid karyotype with 2n = 50 (Figure 1D). Two other cell lines had a near-diploid karyotype.

**Figure 2:**
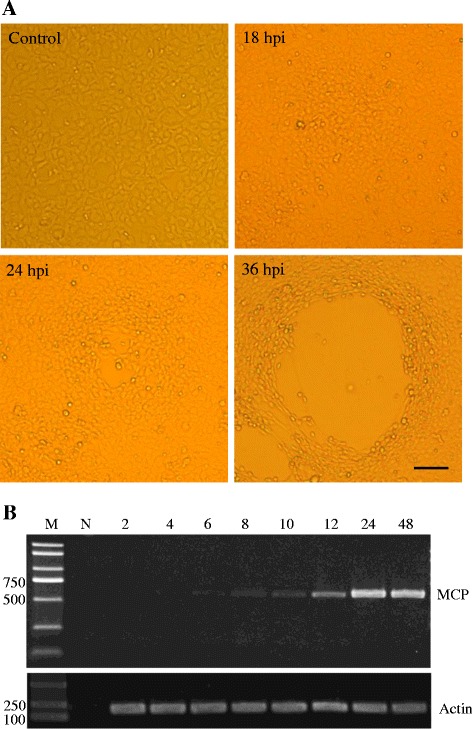
**Microscopic observation and RT-PCR analysis.** **(A)** Micrographs of ADRV-infected GSTC cells. Mock-infected (Control) and infected GSTC cells at 18, 24 and 36 hpi were photographed. Bar = 200 μm. **(B)** RT-PCR analysis of temporal transcription dynamics of MCP gene in ADRV-infected GSTC cells. Samples were taken from mock-infected (N) and infected GSTC cells at time points of 2, 4, 6, 8, 10, 12, 24 and 48 hpi. Beta-actin (Actin) was used as a positive control.

To test the susceptibilities of GSTC, GSSC and GSKC cells to ADRV, three cell lines were infected with the virus respectively. Cells were seeded into 6-well plates at a density of 6 × 10^5^ cells/well in TC199 medium containing 5% FBS and 20 mmol/L *N*-2-hydroxyethylpiperazine-*N*-2’-ethanesulfonic acid. After incubation overnight at 25 °C, the cells were challenged with ADRV at multiplicities of infection of 0.01. The virus was removed after absorption for 1 h, and then 1 mL fresh medium was added. Cytopathic effects (CPE) caused by viruses were observed daily under an inverted light microscope (Leica Microsystems). The supernatant was harvested at 7 days post infection and used for virus titration study as described previously [27]. The viral titer of ADRV in GSTC cells was detected (10^8.4^TCID_50_/mL), which was higher than that in GSSC cells (10^8.0^ TCID_50_/mL) and GSKC cells (10^7.6^ TCID_50_/mL). The development of CPE in ADRV-infected GSTC cells was persistently observed. The infected cells exhibited localized morphological changes such as cell shrinkage and aggregation at 18 hours post infection (hpi). As CPE developed, the aggregated cells became disintegrated and clear CPE were visible at 24 hpi. The infected cells were degenerated and detached, and plaques could be observed at 36 hpi. However, there was no CPE in mock-infected cells all the time (Figure 2A). To confirm the susceptibility of the cells to ADRV further, reverse transcription polymerase chain reaction (RT-PCR) was performed to study the temporal pattern of ADRV major capsid protein (MCP) transcription. Total RNA were extracted from mock-infected or infected GSTC cells at 2, 4, 6, 8, 10, 12, 24 and 48 hpi as we previously did. A pair of primers targeted to the MCP gene (forward primer: 5’TCTCTGGAGAAGAAGAA3’; reverse primer: 5’GACTTGGCCACTTATGAC3’) [11] was used for PCR. The PCR program was carried out as follows: 4 min at 94 °C and then 30 s at 94 °C, 30 s at 50 °C, 45 s at 72 °C for 32 cycles, followed by 72 °C for 5 min. PCR products were sequenced and compared with the MCP sequences of ADRV. As shown in Figure 2B, a specific 532 bp DNA fragment could be amplified in ADRV-infected GSTC cells from 6 hpi, and the sequences were 100% identical to the corresponding nucleotide sequences from ADRV. The expression of MCP gene increased from 6 to 24 hpi, while the identical band could not be detected in the uninfected cells or infected cells at 2 and 4 hpi (Figure 2B). The combination of microscopic observation and RT-PCR analysis showed that GSTC cells can support the wild-type ranavirus, *Andria davidianus* ranavirus (ADRV) replication.

**Figure 3:**
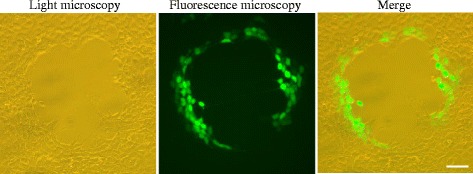
**Light microscopy and fluorescent microscopy observations of rRGV-infected GSTC cells.** Infected GSTC cells not only showed CPE, but also emitted a green fluorescence signal. Bar = 100 μm.

The recombinant *Rana grylio* virus carrying enhanced green fluorescent protein (rRGV-EGFP) was constructed by inserting the EGFP gene into 67R gene locus of RGV as described previously [18]. GSTC cells were infected with rRGV-EGFP as described above. The CPE was illustrated using a combination of light and fluorescence microscopy (Leica Microsystems). Infections with recombinant ranavirus rRGV-EGFP and wild-type ranavirus ADRV induced similar cytopathic characteristics under light microscopy. In addition, the rRGV-EGFP-infected cells emitted strong green fluorescence signal under fluorescence microscopy (Figure 3). Thus the infection process of rRGV-EGFP in GSTC cells can be directly observed by an alternative way. Our study suggests that GSTC cells are sensitive host cells for recombinant RGV replication.

**Figure 4:**
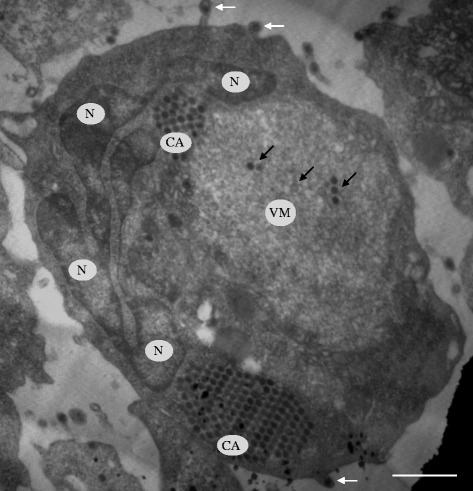
**Electron microscopy observations of ADRV-infected GSTC cells.** Electron micrograph shows the deformed nucleus (N) and low electron density viromatrix (VM) with small or large crystalline arrays (CA) of ADRV viral particles. Some viral particles were budding from GSTC cell (white arrows). A few scattered viral particles were observed within VM (black arrows). Bar = 1 μm.

For electron microscopy observation, ADRV-infected GSTC cells were harvested after appearance of 20% CPE, and trypsinized with 0.25% trypsin prior to centrifugation. The pellet was used for subsequent electron microscopy as we previously did [24]. The ultra thin sections were double stained with saturated aqueous uranyl acetate and lead citrate, and examined with an electron microscope (JEM-1230, JEOL, Tokyo, Japan). Electron micrograph of ADRV infected cell shows severe deformation of the nucleus (N), viromatrix (VM) regions with low electron density, small or large crystalline arrays of virus particles (CA) outside the viromatrix, and only a few scattered viral particles within viromatrix. In addition, budding of ADRV particles from the plasma membrane of infected cells were also seen (Figure 4).

## Discussion

In this study, three cell lines from the Chinese giant salamander, namely the thymus cell line (GSTC), spleen cell line (GSSC), and kidney cell line (GSKC) were developed. Among them, GSTC is the first reported cell line that was established from amphibian thymus tissue, the central immune organ of amphibians [28]. More than 280 cell lines have been established from fishes [24], while there has been no report about a permanent cell line from the Chinese giant salamander. Although most of the established amphibian cell lines grow well in L15 medium [25,26], Chinese giant salamander primary cells failed to be cultivated in L15 or MEM medium. We finally chose the TC199 medium which is usually used to cultivate fish cell lines. Additionally, it takes more time for primary cells to migrate from Chinese giant salamander tissues (about a month) than fish primary cells (about one to two weeks) [24,27]. This is likely to be the reason that the Chinese giant salamander cells take longer to adapt in TC199 medium. The chromosome number in GSTC cells ranged from 28 to 102 with asymmetrical distribution, and the modal number was 50, which occupies 44% in the 100 metaphase cells.

In this study, the sensitivities of three cell lines to ADRV were compared by examining cytopathic effects and viral propagations (virus titers). The appearance of CPE in GSTC cells was faster (24 hpi) and more significant than that in GSSC and GSKC cells (appearing at 36 hpi, data not shown). The highest titer of ADRV (10^8.4^TCID_50_/mL) was also detected in GSTC cells at 7 days post infection. Collectively, these results suggest that GSTC cells are most sensitive to ADRV. This research also shows that the rRGV-EGFP expressed green fluorescent protein after infecting GSTC cells, which provides a convenient way to observe the infection site and development of CPE. It suggests that the GSTC was suitable for recombinant RGV in vitro infection and gene expression. In addition, these cell lines have been used to analyse interactions between ranavirus and the host, the expression profiles and antiviral activity of amphibian immune genes as we have reported before, which confirms that the infection of GSTC cells with ADRV could induce up-regulations of major histocompatibility complex (MHC) isoforms, IFN-inducible protein 6 (IFI6) and T cell receptor beta chain (TCRβ) [29,30].

In summary, we developed three cell lines from the Chinese giant salamander, and the sensitivities of the three cell lines to amphibian ranaviruses were studied. The results show that both wild-type ranavirus ADRV and recombinant ranavirus rRGV-EGFP can replicate and cause CPE in their infected cells, and GSTC has the highest sensitivity to ADRV. Our work provides useful tools for the future study of ADRV, such as identifying ADRV-specific receptors, elucidating the infection mechanism, ADRV recombination and host-pathogen interaction research.

## Abbreviations

GSTC: Chinese giant salamander thymus cell line
GSSC: Chinese giant salamander spleen cell line
GSKC: Chinese giant salamander kidney cell line
ADRV: *Andria davidianus* ranavirus
rRGV-EGFP: recombinant *Rana grylio* virus carrying enhanced green fluorescent protein
MCP: Major capsid protein
FBS: Fetal bovine serum
CPE: Cytopathic effect
hpi: hours post infection
RT-PCR: reverse transcription polymerase chain reaction
